# A Systematic Review of Outcome Measurements and Quality of Studies Evaluating Fixed Tooth-Supported Restorations

**DOI:** 10.1111/jopr.12160

**Published:** 2014-06-19

**Authors:** Devangkumar Rajnikant Patel, Tim O'Brien, Aviva Petrie, Haralampos Petridis

**Affiliations:** 1 Prosthodontics Unit, Department of Restorative Dentistry, UCL Eastman Dental InstituteLondon, UK; 2 Biostatistics Unit, UCL Eastman Dental InstituteLondon, UK

**Keywords:** Success, survival, treatment failure, complications, standardized criteria, treatment outcome, evidence-based dentistry

## Abstract

**Purpose:**

The purpose of this systematic review was to review clinical studies of fixed tooth-supported prostheses, and to assess the quality of evidence with an emphasis on the assessment of the reporting of outcome measurements. Multiple hypotheses were generated to compare the effect of study type on different outcome modifiers and to compare the quality of publications before and after January 2005.

**Materials and Methods:**

An electronic search was conducted using specific databases (MEDLINE via Ovid, EMBASE via Ovid, Cochrane Library) through July 2012. This was complemented by hand searching the past 10 years of issues of the *Journal of Oral Rehabilitation*, *Journal of Prosthetic Dentistry*, *Journal of Prosthodontics*, and the *International Journal of Prosthodontics*. All experimental and observational clinical studies evaluating survival, success, failure, and complications of tooth-supported extracoronal fixed partial dentures, crowns, and onlays were included. No restrictions on age or follow-up time were placed.

**Results:**

The electronic search generated 14,869 papers, of which 206 papers were included for full-text review. Hand-searching added 23 papers. Inclusion criteria were met by 182 papers and were included for the review. The majority were retrospective studies. Only 8 (4.4%) were randomized controlled trials. The majority of the studies measured survival and failure, and few studies recorded data on success; however, more than 60% of the studies failed to define survival, success, and failure. Many studies did not use any standardized criteria for assessment of the quality of the restorations and, when standardized criteria were used, they were modified, thereby not allowing for comparisons with other studies. There was an increase of 21.8% in the number of studies evaluating outcome measurements of all-ceramic restorations in past 8 years.

**Conclusions:**

Prosthodontic literature presents with a reduced percentage of RCTs compared to other disciplines in dentistry. The overall quality of recording prosthodontic outcome measurements has not improved greatly in the past 8 years.

Clinicians are confronted daily with treatment dilemmas for their patients. Ideally, informed treatment decisions should be based on sound scientific evidence, combined with patient desires and clinical experience.^1^ Therefore, it is important that clinicians recognize good-quality evidence and only use such studies in support of their daily practice. Treatment outcome measurements are a key factor in evaluating various treatment modalities. These are often expressed in published studies with various terms such as “survival,” “success,” “failure,” and “complications.” Studies assessing various outcome measurements should be conducted using standardized methods, and all important aspects of the studies should be reported clearly to maintain transparency and reduce the risk of bias.^2^,^3^ Therefore, the quality of study design and reporting of results are very important aspects when the literature is used to compare treatment modalities and prioritize treatment options. These factors have been recognized by both medical and dental researchers, and the quality of published studies of various disciplines has been assessed in both fields.^4^–^13^

Numerous papers have evaluated fixed prosthodontic treatment outcomes; however, the definitions of success and survival, and the criteria used to evaluate the data differ greatly among different studies. These variations in definitions hinder the interpretation and reliable combination of data over several studies and may preclude any meaningful direct comparisons of fixed prosthodontic treatment outcomes.^2^,^11^,^14^,^15^ Pjetursson et al^16^ considered a fixed partial denture (FPD) to be successful if it remained unchanged and free from all complications over the entire observation period. An FPD was considered to have survived if it remained in situ with or without modification over the observation period. The most objective category of failure is the removal of the FPD; however, this definition of failure clearly overstates the success of FPDs, as many are found in situ but in need of replacement.^17^ A recent systematic review^18^ of the literature identified such issues in a large number of implant-related studies. Most studies were unclear regarding the nature of the study groups and failed to clearly define “survival” and “success;” the terms were often used interchangeably. Torabinejad et al^11^ observed that methods for calculating outcomes were not always reported in studies related to FPDs and complications were mostly not described. Lack of reporting quality in systematic reviews and RCTs has also been shown in studies evaluating various dental disciplines.^9^,^10^,^13^ A recent study^12^ showed a slight improvement in quality of reporting in implant dentistry over the past few years; however, most of the literature was still of low quality. Another study^9^ assessing the quality of reporting of systematic reviews in orthodontics compared reviews published between 1999 and 2004 and 2004 and 2009 and concluded there was no significant improvement in the quality of reporting.

Recommendations on reporting have been made for studies assessing longevity of restorations in order to standardize the measurement of outcomes.^2^ Clinical indices such as USPHS/Ryge criteria,^19^ CDA criteria,^20^ and Hickel's criteria^21^ have been developed, in an attempt to standardize the criteria for evaluating restorations. These criteria assess restoration qualities such as anatomic form, marginal integrity, caries, and color. According to the USPHS criteria^19^ all categories are given five scores: Alfa, Bravo, Charlie, Delta, and Oscar to determine if the restoration is in an excellent state or failing. In the CDA criteria^20^ satisfactory restorations are divided into “excellent” (A) and “acceptable” (B), and nonsatisfactory restorations are divided into “correct/replace” (C) and “replace” (D). Modifications of USPHS and CDA criteria have been used to include more categories for assessment. The proper use of such criteria has a direct effect on the reporting of outcome measurements. Moreover, forms have been developed for all-ceramic restorations to describe and classify details of chipping fractures and bulk fractures to satisfy criteria for comprehensive failure analyses and subsequent treatment planning.^22^ Nevertheless, some authors argue against using USPHS or CDA criteria because the degree of deviation from the ideal state is recorded on its own without considering other factors, and results may not be applied with validity to different clinical circumstances.^3^

There is no current comprehensive information available regarding the quality of study design, method of recording, and reporting of outcome measurements in studies evaluating tooth-supported fixed prostheses. The purpose of this systematic review was to review clinical studies of tooth-supported fixed prostheses, and assess study design, as well as the quality of recording and reporting of outcome measurements. Multiple hypotheses were generated to explore associations among various factors related to study design and different outcome modifiers and to compare the quality of the literature published prior to and after January 2005. This cut-off date was chosen arbitrarily, according to a similar article^9^ looking at the orthodontic literature.

## Materials and Methods

### Search methods for identification of studies

#### Electronic search

Electronic literature searches of medical databases (Cochrane Database of Systematic Reviews and RCTs [1980 to July 2012], EMBASE [1974 to July 2012], and MEDLINE [1948 to July 2012]), were carried out by one reviewer (DP) using specific search strategies, which included combination of MeSH terms and keywords: ((Crown$.mp. OR exp Crowns/ OR Onlay$.mp. OR Fixed partial denture.mp. OR exp Denture, Partial, Fixed/ Bridge$.mp.) *AND* (exp Prosthesis Failure/ OR exp Dental Restoration Failure/ OR exp Treatment failure/ OR Postoperative Complications/ OR Complication.mp. OR Success.mp OR Survival/ OR survival.mp. OR Longevity.mp. OR exp. Longevity OR Outcome adj3 Measur$)).

#### Searching other resources

Hand searching for relevant studies was performed by DP for the past 10 years of issues of the following journals: *Journal of Oral Rehabilitation* (JOR), *Journal of Prosthetic Dentistry* (JPD), *Journal of Prosthodontics* (JP), and *International Journal of Prosthodontics* (IJP).

### Selection of studies

The selection process was conducted in two phases. During the first phase the titles and abstracts were screened by one reviewer (DP) according to the following inclusion and exclusion criteria:

#### Type of intervention

Clinical studies on humans, evaluating extracoronal tooth-supported restorations, specifically crowns, FPDs, and onlays were included, irrespective of material used. Studies exclusively related to implant-supported restorations, implant/tooth-supported or removable prostheses, post and core restorations, veneers and resin-retained FPDs, as well as studies evaluating intracoronal restorations were excluded.

#### Types of studies

Both observational studies (prospective cohort studies, retrospective cohort studies, case control studies, and cross sectional studies) and experimental studies (randomized and nonrandomized controlled trials, noncontrolled clinical trials, and case-series) were included for the review. Studies involving questionnaires and interviews, clinical reports, and expert opinion papers were excluded. Only studies published in English were included.

#### Follow-up time

As the identification of outcome measurements was one of the primary goals of this study, all studies were considered for inclusion irrespective of their follow-up time.

#### Types of outcome measurements

Clinical studies evaluating quantitative outcome measurements such as success, survival, failure and/or complications were considered for inclusion. If a study included both relevant and irrelevant data, for example, implant- and tooth-supported restorations, it was included, but only the relevant data was used towards the final review.

Another reviewer (TOB) independently screened the results. Any disagreements were resolved by discussion between three reviewers (DP, HP and TOB) and in cases where there was any doubt, the full text of the paper was obtained. The full text of all the papers that passed the first review phase was finally retrieved. Full-text papers generated from hand searching were added after discussion for any disagreement. The second phase of the review included all full-text papers, which were assessed by two reviewers (DP and TOB) for inclusion or exclusion by applying the criteria set previously. A specific data collection form was constructed, and a pilot run was performed. Any disagreements between DP and TOB were resolved via discussion and refereeing by HP. Data collection of all included papers was done by one reviewer (DP). Regarding data collection, inter-reviewer agreement between TOB and DP was calculated by assessing 10% of the papers. DP further reviewed 10% of the papers, again, for intrareviewer agreement calculation to be performed. Inter-reviewer and intra-reviewer agreement were determined using Cohen's kappa (κ) coefficients.^23^

### Data collection and analysis

Once the final number of included studies was agreed upon by three of the authors (DP, HP and TOB), data were collected using the custom data collection form and set guidelines for each of the following four categories: demographic data, clinical data, study design, and outcome measurements recording and reporting.

### Statistical analyses of hypotheses

Multiple hypotheses were generated to explore associations among various factors related to study design and different outcome modifiers and to compare the quality of literature published between two time periods: from 1948 to December 2004 (group 1) and from January 2005 to July 2012 (group 2). Once all the data were collected, frequencies and percentages were analyzed and compared by the Pearson's Chi-Square test using statistical software SPSS v 17.0 for windows (SPSS Inc, Chicago, IL). The level of significance was set at 0.01 rather than the conventional 0.05 to avoid spuriously significant results arising from multiple testing.

## Results

**Figure 1:**
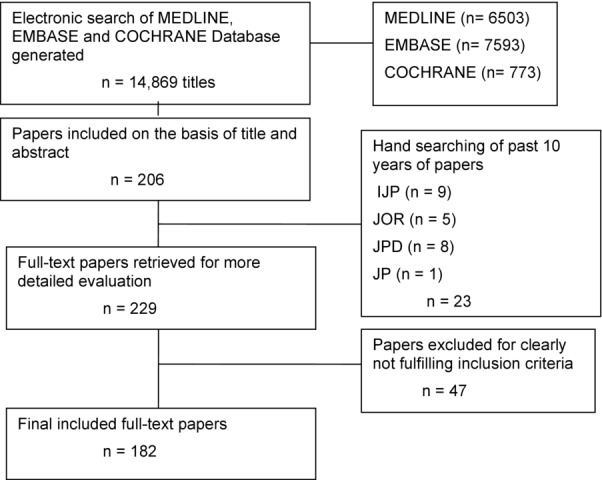
Flow chart of results generated by search strategy and final result after application of inclusion/exclusion criteria.

The study selection flow chart is illustrated in Figure 1. The electronic database search generated 14,869 papers. After title and abstract reviewing, 206 papers were included for full-text retrieval. Journal hand searching produced 23 more full-text papers. Phase 2 review of the full texts led to the exclusion of 47 additional studies (reasons for exclusion are depicted in Table 1), resulting in 182 papers included for the final analysis. Included studies are listed in the Appendix. There was good inter-reviewer (inclusion/exclusion κ = 0.91; data extraction κ = 0.82) and intra-reviewer agreement (κ = 0.95) during all selection phases.

**Table 1:** Number of excluded studies during phase 2, and reasons for exclusion

Details of excluded studies	
Reason for exclusion	Number of studies
Same cohort used in different studies	12
No success, survival, failures, or	12
complications measured	
Resin-retained FPD, intracoronal restorations	10
Clinical reports	4
Descriptive study, literature review	7
In vitro study	2

### Demographic data

Results showed that all studies had recorded information on the final number of patients. However, age range and mean age of the patients were not recorded by 31.3% and 40.7% of studies, respectively.

### Clinical data

The majority of studies (85.7%) reported some information on tooth descriptors, mostly the position of the tooth in the mouth (76.9%); however, less than half of those studies reported the periodontal (38.5%) or endodontic status (41.8%) of the teeth. Very few studies (8.2%) reported on the amount of remaining tooth structure prior to restoration placement.

The majority of included studies (65.4%) did not provide any information on certain patient characteristics, such as the type of occlusion or the presence of parafunction. The reasons for restoration placement were usually not (85.7%) provided.

The most frequent restorations studied were full-coverage crowns (34.6%) and FPDs (60.9%). In most included studies (72%) the type of luting agent was described, and in almost half (51.6%) the tooth preparation and finish was explained. The most commonly evaluated restoration materials were all-ceramic (58.8%), followed by metal-ceramic (29.7%). Most of the included studies had been conducted in a university setting (57.1%), whereas 27.5% had used private practice settings.

### Study design

Most of the included studies were observational (57.7%), and only 4.4% were RCTs (Table 2).

**Table 2:** Frequency (percentage) of types of studies included for review

Type of study	
Study type	Frequency (%)
**Observational study**	**105 (57.7%)**
Retrospective cohort study	99 (54.4%)
Prospective cohort study	6 (3.3%)
**Experimental study**	**77 (42.3%)**
RCT	8 (4.4%)
Controlled clinical trial (non-randomized)	3 (1.6%)
Clinical trials (uncontrolled, non-randomized)	66 (36.3%)
**Total**	**182 (100.0%)**

### Outcome measurements recording and reporting

#### Survival, success, and failure

Survival was measured in 135 studies; however, only 23 defined the term “survival.” Success was measured in 65 studies, but only 26 defined the criteria for “success.” Failure was measured in 152 studies, and only 54 defined the term ‘’failure’’ (Table 3).

**Table 3:** Frequency of studies recording data on survival, success, and failure

Data on survival, success, and failure			
Category	Yes frequency (%)	No frequency (%)	Total frequency (%)
Survival measured	135 (74.2%)	47 (25.8%)	182 (100%)
Survival defined	23 (17.0%)	112 (83.0%)	135 (100%)
Success measured	65 (35.7%)	117 (64.3%)	182 (100%)
Success defined	26 (40.0%)	39 (60.0%)	65 (100%)
Failure measured	152 (83.5%)	30 (16.05%)	182 (100%)
Failure defined	54 (35.5%)	98 (64.5%)	152 (100%)

Various definitions of “survival” were provided, such as:

1. “Restored tooth remained intact, fixed prosthesis remaining intact, restored tooth remaining free from radiographic and clinical signs and symptoms of pulp deterioration.”^24^
2. “The period of time starting at the cementation of the restoration and ending when the crown was shown to have irreparably failed, for example, Porcelain fracture or partial debonding that exposed the tooth structure and impaired aesthetic quality.”^25^
3. “Crown not removed.’’^26^

Similar variation could be found in the definition of “success” among the included studies:

1. “Those crowns that were present without core fracture, porcelain fracture, caries, sign of periodontal inflammation (specifically bleeding on probing), or endodontic sign and symptoms.”^27^
2. “Restorations still in clinical service.” 28
3. “No framework fracture of zirconia.”^29^

Various definitions of “failure” were:

1. “Any defects in its design or execution which necessitated the remaking of the bridge.”^30^
2. “Need for replacement.”^31^
3. “Crown or tooth loss.”^32^

#### Types of methods used for survival, success, and failure analyses

It was disturbing to find that more than a third of included studies (37.7%) had not described the method of statistical analysis of survival data. Among the studies that did report the statistical methods, Kaplan-Meier analysis was the most popular (55.4%).

#### Complications

The majority (94.5%) of included studies reported data encompassing a wide range of biological, mechanical, and esthetic prosthesis complications (Table 4). Other types of complications including sensitivity, mobility, overcontoured crown, temporomandibular dysfunction, dislodgement of the post, etc. were recorded by 44.8% of the studies.

**Table 4:** Frequency of studies recording data on various types of complications

Complication type	Frequency (%)	Complication type	Frequency (%)	Complication type	Frequency (%)	
Biological	157 (91.3%)	Mechanical	168 (97.7%)	Esthetic	108 (62.8%)	Other complications
Pain	28 (16.3%)	Porcelain fracture	153 (89.0%)	Poor esthetics	106 (61.6%)	77 (44.8%)
Caries	136 (79.1%)	Fractured tooth/root	98 (57.0%)	Recession	12 (7.0%)	
Periapical pathology	115 (66.9%)	Fractured prostheses	149 (86.6%)			
Periodontal disease	100 (58.1%)	Loss of retention	97 (56.4%)			
Effect on opposing tooth	11 (6.4%)	Defective margins	105 (61.0%)			

#### Standardized criteria

More than half of the studies did not use any standardized criteria for prosthesis evaluation (Table 5).

**Table 5:** Frequency of criteria used to assess the quality of restorations

Standardized criteria	
Criteria used	Frequency (%)
CDA Criteria	31 (17%)
Modified CDA Criteria	12 (6.6%)
USPHS Criteria	6 (3.3%)
Modified USPHS Criteria	31 (17%)
Other	6 (3.3%)
None	96 (52.8%)
Total	182 (100.0%)

### Results of the hypotheses testing

Detailed results from hypotheses testing are provided in Table 6.

**Table 6:** Results of null hypotheses testing

Results of null hypotheses testing (significance level *p* < 0.01)			
Null hypotheses	*p* Value	Observational study (n/total)	Experimental study (n/total)
Study design is not associated with the fact that success, survival, and failures were well defined	0.956 (Survival)	13/77	10/58
	0.415 (Success)	16/36	10/29
	0.006 (Failure)	40/90	14/62
Study design is not associated with the lack of use of analytical methods for assessment of survival, success, and failure	0.583	36/105	29/77
Study design is not associated with the type of setting used (private practice vs university)	0.213	34 (Private) & 64 (University) /98	16 (Private) & 47 (University) /63
Study design is not associated with the patient descriptors	0.003	78/105	41/77
Study design is not associated with the tooth descriptor	<0.001	23/105	3/77
Study design is not associated with the description of tooth preparation included in the paper	<0.001	28/105	61/77
Study design is not associated with the type of material used	<0.001 (all-ceramic)	42/105	65/77
	<0.001(PFM)	44/105	10/77
Study design is not associated with the use of the standardized criteria to assess restoration	<0.001	32 /105	54 /77
		Group 1(n)/ Total	Group 2(n)/ Total
The proportions of recording definitions for fixed prosthodontic outcomes (i.e., survival, success, and failure) is the same for groups 1* and 2**	0.267 (Survival)	6/48	15/74
	0.156 (Success)	10/32	16/33
	0.339 (Failure)	24/76	30/77
The proportions of reporting of methods used to analyze outcomes is the same for groups 1 and 2	<0.001	44/83	21/91
The proportions of type of setting used is the same for groups 1 and 2 (private practice vs. university)	0.020	49/81	62/80
The proportions of reporting patient descriptors is the same for groups 1 and 2	0.326	62 /90	57 /92
The proportions of reporting tooth descriptors is the same for groups 1 and 2	0.002	20/90	6/92
The proportions of reporting detailed tooth preparation is the same for groups 1 and 2	0.005	37/90	57/92
The proportions of using different types of restorative materials is the same for groups 1 and 2	0.003 (all-ceramic)	43/90	64/92
	0.923 (PFM)	27/90	27/92
The proportions of reporting the use of standardized criteria is the same for groups 1 and 2	0.453	40/90	46/92
The proportions of experimental studies and observational studies measuring fixed prosthodontics outcomes is the same for groups 1 and 2	0.128	33 (experimental) & 57 (observational)/90	44 (experimental) & 48 (observational) /92

* Group 1 (Literature published up to the end of December 2004).

** Group 2 (Literature published from January 2005 until July 2012).

#### Relation between study design and important factors related to outcome measurements

There was a significant effect (*p* < 0.01) of study design, that is, observational or experimental, on type of material used, use of standardized criteria to evaluate restorations, description of tooth preparation and lack of information on patients and tooth descriptors. Observational studies evaluated more metal-ceramic restorations than all-ceramic restorations. This category also used standardized criteria less frequently, and there was a lack of detailed record of tooth preparation and patient and tooth descriptors, whereas experimental studies performed significantly better in those areas. Observational studies recorded definition of failure significantly more frequently than experimental studies; however, there was no statistically significant effect (*p* > 0.01) of study design and frequency of recording data on the definition of survival and success or on the type of settings used.

#### Comparison between studies published until December 2004 (group 1) and from January 2005 till July 2012 (group 2)

Group 1 consisted of 90 papers, and group 2 consisted of 92 papers. Generally papers included in group 2 recorded relevant information more frequently than papers included in group 1; however, the difference was not statistically significant in most categories.

#### Statistically significant (*p* < 0.01) differences were found for the following issues (Table 6)

In group 1, 22.2% of the papers did not record any type of tooth descriptor, but an improvement was seen in group 2 studies where only 6.5% failed to record any type of tooth descriptor. More studies in group 2 described type of tooth preparation undertaken and luting cement used than in group 1. Group 2 recorded detailed tooth preparations significantly more frequently than group 1. An increase of more than 20% in the papers evaluating all-ceramic crowns was noted in group 2 studies. In group 2, 10.9% of the studies failed to record the type of setting used, whereas only 4.4% of the studies failed to record this information in group 1. Group 1 described the method of analysis used to assess survival, success, and failure significantly less frequently then group 2.

## Discussion

There is a lack of data available on the quality of recording and reporting outcome measurements in tooth-supported fixed prostheses. Furthermore, there is a lack of evidence showing any improvements in reporting outcome measurements of fixed prostheses in recent years. The purpose of this systematic review was to review clinical studies of tooth-supported fixed prostheses, and assess study design as well as the quality of recording and reporting of outcome measurements. A broad literature search was conducted to avoid missing any important papers. The inclusion and exclusion criteria were clearly defined and peer-reviewed to help generate the required papers for assessment.

The results of this systematic review showed that some baseline information, such as the reason for restoration of the tooth, and patient descriptors including bruxism, was not always recorded accurately. This finding is in agreement with a previous study^13^ that looked at the quality of reporting of RCTs in prosthodontics, a study^2^ published on direct restorations, and a recent review^18^ of outcome measures in implant literature. Bruxism has been associated with porcelain chipping and increased frequency of complications;^33^ therefore, the lack of reporting of this and other prognostic factors or patient characteristics as confounding variables may significantly affect the results.

The results of this study also showed there has been no significant increase in the percentage of RCTs in published studies pertaining to tooth-supported fixed restorations through the years. This is in contrast to literature that has evaluated the percentage of published RCTs in implant^18^ and dental^7^ literature.

The primary outcomes evaluated in this review were survival, success, failures, and complications. To maintain transparency and comparability, it is crucial to record the details of measured outcomes with criteria against which they were assessed. Generally survival of restorations relates to the restorations being in situ irrespective of their condition, whereas usually criteria measuring success are stricter. Hence, it is impossible to compare studies measuring success with studies measuring survival. It is also not feasible to compare two studies measuring success of the same type of restorations if the definition of success is different. A variety of definitions of similar outcomes, that is, survival, success, or failure, were used, and not all authors adhered to the same strict criteria in the included studies. This finding has been reported by other studies as well.^14^,^18^ Reduced numbers of definitions confirms the lack of standardization of the measurements and design between studies.^11^ Use of standardized definitions of survival, success, and failure is also recommended.

While calculating the survival time of a particular restoration, some difficulty may occur due to patients being lost to follow-up or due to the study terminating prior to the patients experiencing the outcome event. This phenomenon is known as censoring. If censoring is not taken into account, then survival data may underestimate the true time to event analysis, and hence special methods are needed to evaluate survival, and standard methods of data exploration may not be useful.^34^ The results of this study showed that 38.9% of the studies did not record the type of method used to evaluate the survival data. This finding is in agreement with another recent study^35^ and has also been reported in a review^18^ of implant literature.

Understanding relevant complications related to fixed prosthodontics can improve the clinician's ability to perform accurate diagnosis, construct an appropriate treatment plan, set realistic expectations for the patients, and plan postoperative care. Using set criteria for evaluating the quality of restorations can produce more consistent and comparable results for particular outcome measurements. The results of this study showed that the evaluation process of quality of restorations still a lacks standardization, since half the studies did not use any standardized indices to evaluate the quality of restorations. Some studies assessed occlusion and the level of interference along with other factors using USPHS criteria.^36^ One study assessed only marginal quality, contour, surface texture, and color match,^37^ whereas another assessed postoperative sensitivity, recurrent caries, marginal adaptation, proximal contact, anatomic form, and surface texture.^38^ Both of these studies used USPHS criteria but modified them differently. Many studies used modified USPHS criteria; however, there was lack of standardization among assessment procedures, which is in agreement with other studies.^2^,^3^

Multiple hypotheses were generated to assess the relationship between study design and different factors, such as methods of reporting, definitions, and assessment criteria. Fundamentally, observational studies, especially retrospective cohort studies, are different than experimental studies and limited by the type of data recorded at the time of initial examination, at the time of treatment, and on the last examination in order to assess the restoration. This review identified most of the studies as retrospective, and various criteria had possibly not been defined at the time of the intervention. The analysis of the results of this study showed that the type of study had no significant influence on the frequency that the definition of survival and success was recorded, and no influence on the type of method used for outcome analysis; however, experimental studies generally recorded more information and were more standardized than observational studies. This result further emphasizes the need for and reflects the robustness of well-designed experimental studies.

In the past two decades evidence-based dentistry has become more popular, and various tools have been generated to assess the quality of reporting and, ultimately, to improve the quality of future studies.^39^,^40^ In this study, an attempt was made to compare various factors between studies published before (group 1) and after (group 2) the end of 2004. Evaluation of studies published between groups 1 and 2 showed an improvement in reporting of methods of analysis used; however, there was no significant improvement in defining success, survival, and failure or use of standardized criteria. This result meant that even in prospective trials, the quality of recording this information has not improved. Hence, better constructed prospective clinical trials are recommended for future research. The importance of blinded randomized controlled trials or adequately conducted prospective trials has been stressed in recent years to improve the quality of the studies.^41^ However, the results of this study showed no statistical difference between the proportions of experimental and observational studies published in recent years. This may suggest various limitations in conducting intervention studies for tooth-supported fixed restorations due to various reasons (e.g., ethical approval). This finding is not comparable to any other study. This systematic review was in agreement with other studies^9^,^10^,^18^ published in various other disciplines of dentistry, showing no significant improvement in the quality of published literature and overall quality of recording outcome measurements in recent years.

This study has limitations, such as the exclusion of non-English and grey literature, the fact that citations and references of included papers were not searched to find more articles, and that only 10% of the total papers were reevaluated at the data-collection stage; however, sensitive electronic and manual searches, coupled with the large number of final papers included, probably outweighed the above limitations. This was reflected by the satisfactory inter-reviewer and intra-reviewer agreement during the reevaluation phase after article search and collection.

Research papers on outcome measurements are very important for clinical day-to-day practice as they may provide clinicians with better understanding for making appropriate treatment decisions. They also provide appropriate and more accurate information in order to gain valid consent from the patients. The combination of the results of multiple research papers, such as in the form of meta-analyses, can provide very robust conclusions. The results of this systematic review showed a lack of standardization between studies evaluating similar outcomes, thus hindering the combination of data. Of particular concern is the lack of standardization regarding definitions of outcomes such as survival, success, and failure. The prosthodontic community should organize a consensus statement with precise definitions for the aforementioned terms which can be used by future studies. The same holds true regarding the standardizations of restoration evaluation criteria as has been previously suggested.^2^ The adherence of researchers to available guidelines such as CONSORT guidelines for RCTs^42^ and STROBE for observational studies^43^ will further enhance the quality and standardization of future published papers.

## Conclusions

Within the limitations of this study the following conclusions could be drawn:

1. There has been no increase in published RCTs in prosthodontics during past decade compared to previous years.
2. A large proportion of the studies had problems with the definition, standardization, or with the reporting of methods used to calculate survival, success, and failure.
3. More than half of the studies did not use any standardized criteria for quality evaluation of the restorations.
4. The overall quality of recording prosthodontic outcome measurements has not improved greatly in past 8 years.
